# Acupuncture for inflammatory bowel disease: A protocol for systematic review and meta-analysis

**DOI:** 10.1097/MD.0000000000032236

**Published:** 2022-12-09

**Authors:** Xinyue Yang, Mengmeng Sun, Min He, Zhihong Wang, Qingqing Tang, Tie Li

**Affiliations:** a Department of Acupuncture and Tuina, Changchun University of Chinese Medicine, Changchun, China; b Northeast Asia Research Institute of Traditional Chinese Medicine, Changchun University of Chinese Medicine, Changchun, China.

**Keywords:** acupuncture, CD, IBD, inflammatory bowel disease, meta-analysis, protocol, systematic review, UC

## Abstract

Inflammatory bowel disease is a prevalent condition that has a major impact on the patient’s life. The conventional drugs for IBD have limits, such as unpleasant events and a difficult recovery. External treatment such as acupuncture, is a traditional Chinese medicine-based therapy in which needles are used to restore the body’s internal balance, and is gaining more and more popularity as a therapeutic option for IBD. However, there is a lack of evidence to support its efficacy and safety in IBD patients. The goal of this systematic review is to assess the evidence of acupuncture’s efficacy and safety for IBD.

**Methods:**

MEDLINE, the Cochrane library, EMBASE, Web of Science, China National Knowledge Infrastructure, the Chongqing VIP Chinese Science, Technology Periodical Database, the Wanfang database, Japanese medical database, Korean Robotics Institute Summer Scholars, and Thailand Thai-Journal Citation Index Centre will be searched from their inception to 9 November, 2022. Randomized controlled trials evaluating the efficacy of manual acupuncture for patients with IBD, whether or not the blind technique is utilized, will be considered. Language and publication time are both unrestricted. Review Manager (V.5.3.5) will be used by 2 separate researchers to perform article retrieval, duplicate removal, screening, quality evaluation, and data analysis. Efficacy and safety of acupuncture for IBD will be assessed using outcomes including as total effective rate or cure rate, clinical symptom integral (abdominal pain, diarrhea, purulent stool), recurrence rate, inflammatory cytokines, and the Baron and Mayo scores.

**Results:**

The protocol of this study systematically will assess the effectiveness and safety of acupuncture for IBD.

**Conclusion:**

This study investigates the efficacy and safety of acupuncture for IBD, providing clinicians and patients with additional options for the treatment of this disease.

## 1. Introduction

Inflammatory bowel disease (IBD) is a chronic relapsing gastrointestinal tract inflammatory disorder that includes ulcerative colitis and Crohn’s disease (CD). It is caused by chronic or relapsing immune activation and corresponding inflammation inside the gastrointestinal tract.^[1,2]^ IBD is estimated to affect around 1 million people in the United States and 2.5 million in Europe. Furthermore, IBD has appeared in newly industrialized countries in Asia, South America, and the Middle East, and has grown into a worldwide illness with increased frequency on every continent.^[3,4]^ In Japan, the number of patients with IBD has been growing since 1950.^[5]^ According to Chinese literature in 20th-century, the total number of IBD cases increased by approximately 2.5-fold over the previous decade, with CD patients increasing by 15.7-fold.^[6]^ There is a need for comprehensive knowledge regarding these diseases since they represent a growing global health burden with difficult-to-manage costs.^[7]^

IBD is still on the rise globally, owing primarily to an increase in newly industrialized countries. Despite significant progress, the precise pathophysiology of IBD remains unknown, several lines of evidence show that the disease is caused by a confluence of environmental, microbial, immunological, and genetic factors.^[8–10]^ The elucidation of the mechanism of IBD has facilitated therapeutic development.^[11]^ Glucocorticoids, amino salicylic acid preparations, immunosuppressive drugs, biological preparations, surgery, and other treatments are now used in western medicine to treat IBD.^[5,12–17]^ As long course of disease, easy relapse, protracted and difficult recovery, seriously affecting the quality of life, increasing the economic burden of patients and society, and even increasing the risk of developing cancer, IBD has become one of the medical field’s hot topics of general concern.^[18]^

External therapy such as acupuncture is based on theories of Traditional Chinese Medicine, which is with easy, safe, effective, and nontoxic side effects. It is gaining popularity as a method of therapy in IBD.^[19–21]^ However, in clinical practice, there is still a dearth of comprehensive investigation of the efficacy and safety for IBD.As a result, in this research, the efficacy and safety of external therapies in the treatment of IBD will be carefully examined and meta-analyzed.

## 2. Methods

### 2.1. Study registration

The protocol for this review has been registered in the PROSPERO network (No. CRD42021257520). This protocol was designed according to the Preferred Reporting Items for Systematic reviews and Meta-Analyses Protocols (PRISMA-P) 2015 Statement.^[22]^

### 2.2. Eligibility criteria

#### 2.2.1. Types of studies.

Randomized controlled trials evaluating the efficacy of manual acupuncture for patients with IBD, whether or not the blind technique is utilized, will be considered. Language and publication time are both unrestricted.

#### 2.2.2. Types of participants.

Patients with a confirmed clinical, endoscopic, and histological diagnosis of IBD, independent of gender, age, or origin of the case, will be included. However, some patients will be excluded even if they satisfy the clinical criteria for IBD, such as pregnant or nursing women, people with serious heart, liver, or lung problems, and those who have had major trauma surgery in the past.

#### 2.2.3. Types of interventions.

Acupuncture alone or in combination with routine treatment as prescribed by guidelines is used in the treatment group. There are no constraints on the equipment, frequency, treatment course, depth of needling, or number of acupuncture sites.

#### 2.2.4. Types of outcome measures.

##### 2.2.4.1. Primary outcome.

The primary outcomes will be evaluation of treatment efficacy, which includes evaluation of clinical treatment efficacy, clinical symptom integral (abdominal pain, diarrhea, purulent stool), and recurrence rate. The clinical effectiveness is based on the consensus view on diagnosis and therapy proposed in 2018 by the IBD group of the gastrointestinal branch of the Chinese medical association,^[23]^ and there are 3 levels: remission, effective, and ineffective. The total effectiveness rate is a percentage that represents the ratio of the total number of mitigators and effective persons to the total number of people. There are 3 forms of illness relapse: occasional (no more than once per year), frequent (twice per year), and persistent (continuous active disease that cannot be relieved).

##### 2.2.4.2. Secondary outcome.

Inflammatory cytokines, as well as the Baron and Mayo scores, are among the other outcomes. Interleukin-6, Interleukin-17, tumor necrosis factor-α, and other inflammatory cytokines are examples. The Baron score, which ranges from 0 to 4, indicates the harshness of the mucosa during a colonoscopy. The Mayo score employs a scale of 0 to 3 to indicate a change in symptoms such as stool frequency, rectum bleeding, flexible proctosigmoidoscopy results, and the physician’s overall assessment. Furthermore, the rate of adverse reactions is a critical predictor of safety.^[24]^

### 2.3. Searching methods for the identification of studies

Two investigators have independently conducted a literature search from inception in PubMed, the Cochrane Central Register of Controlled Trials, and the Chinese electronic databases, including China National Knowledge Infrastructure, Wan Fang databases, VIP, SinoMed, and the Chinese clinical trial registry, using the 3 key components of intervention method, disease and study type. Taking PubMed as an example, the retrieval strategy is as follows: (“Acupuncture” OR “Acupuncture Therapy” OR “needling” OR “acupressure” OR “Pharmacopuncture” OR “electroacupuncture” OR “Acupuncture Points” OR “Acupuncture Point” OR “Acupoints” OR “Acupoint”) AND (“IBDs” or “Crohn Disease” or “colitis, ulcerative” or “IBD” or “Crohn’s Enteritis “ or “CD” or “Crohns Disease” or “Ulcerative Colitis” or “Colitis Gravis” or “IBD” or “ulcerative colitis” or “CD”) AND (“randomized controlled trials” OR “RCT”), which is showed in Table 1. The Chinese databases were searched using the key words with the same retrieval strategy. A manual search of the reference lists of included papers will enhance the electronic database search. There were no language restrictions, and the last search update has been taken place 9 November, 2022.

**Table 1:** The search strategy for PubMed database.

Number	Search terms
#1	Acupuncture OR Acupuncture Therapy [MeSH]
#2	Acupuncture [Title/Abstract] OR needling [Title/Abstract] OR acupressure [Title/Abstract] OR Pharmacopuncture [Title/Abstract] OR electroacupuncture [Title/Abstract] OR Acupuncture Points [Title/Abstract] OR Acupuncture Point [Title/Abstract] OR Acupoints [Title/Abstract] OR Acupoint [Title/Abstract]
#3	#1 OR #2
#4	Inflammatory Bowel Diseases OR Crohn Disease OR colitis, ulcerative [MeSH]
#5	Inflammatory Bowel Disease [Title/Abstract] OR Crohn’s Enteritis [Title/Abstract] OR Crohn’s Disease [Title/Abstract] OR Crohns Disease [Title/Abstract] OR Ulcerative Colitis [Title/Abstract] OR Colitis Gravis [Title/Abstract] OR IBD [Title/Abstract] OR UC [Title/Abstract] OR CD [Title/Abstract]
#6	#4 OR #5
#7	randomized controlled trial [MeSH]
#8	randomized controlled trial [Publication Type] OR randomized controlled trials as topic [MeSH Terms] OR randomized controlled trial [All Fields] OR RCT [Title/Abstract]
#9	#7 OR #8
#10	#3 AND #6 AND #9

### 2.4. Data collection

#### 2.4.1. Selection of studies.

**Figure 1.**
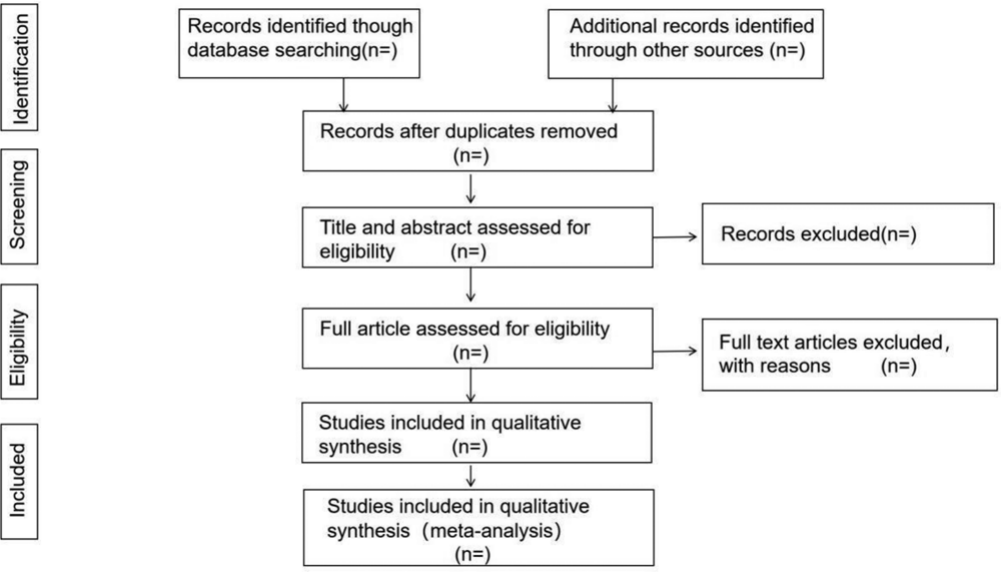
Flow chart of the search process.

To begin, all studies should be imported into Endnote to eliminate duplication. Second, the included publications will be assessed independently by 2 researchers using the research criteria and search methodologies. The whole text of the eligible articles will be read to decide their inclusion. Disagreements will be resolved by discussion with other reviewers during the full-text selection process. Figure 1 depicts the study selection procedure.

#### 2.4.2. Data extraction and management.

Two studies will extract information from the studies that met the inclusion criteria, such as the author, article title, year of publication, participants, intervention measures, comparator, outcome, adverse events, and so on. If the required information is missing, we will contact the original author to obtain the missing information. If the data does not appear to be available, the study will be dismissed.

#### 2.4.3. Assessment of risk of bias.

In this effort, 2 independent researchers will assess the risk of bias in the literature included in the systematic review using the Cochrane Collaboration bias risk assessment tool. Two researchers will analyze bias using the following criteria: random sequence generation, allocation concealment, blinding of participants and personnel, blinding of outcome assessments, insufficient outcome data, selective reporting, and other sources of bias. Each domain’s bias risk will be graded as “low risk,” “high risk,” or “unclear risk.”

### 2.5. Data analysis

#### 2.5.1. Data synthesis.

The RevMan V.5.3.5 software (the Cochrane Collaboration, Oxford, England) will be used to conduct the meta-analysis of intervention and outcome measurement methods in accordance with the statistical guidelines referenced in the current version of the Cochrane Handbook for Systematic Reviews of Interventions. If the statistical heterogeneity is not significant (*P* > .1, or *I*^2^ < 50%), we will use the fixed effect model to combine the data, however if it is significant (*P* < .1, or *I*^2^ > 50%), we will use the random effect model. If the heterogeneity is significant, a narrative, qualitative summary will be undertaken.

#### 2.5.2. Assessment of heterogeneity.

The Chi^2^ test (significance level: 0.1) and *I*^2^ statistic will be used to assess statistical heterogeneity (0%–40%: might not be important; 30%–60%: may represent moderate heterogeneity; 50%–90%: may represent substantial heterogeneity; 75% to 100%: considerable heterogeneity). If there is significant heterogeneity among trials (*I*^2^ ≥ 50% or *P* < .1), the research design and characteristics are included.

#### 2.5.3. Subgroup analysis and sensitivity analysis.

Subgroup and sensitivity analyses will also be carried out to investigate the various sources of heterogeneity. If the necessary data are available, subgroup analyses will be performed for the various acupuncture interventions (e.g., acupuncture/electro-acupuncture vs acupuncture/electro-acupuncture combined with 5-aminosalicylic acid), different types of control groups (western medicines with or without sham acupuncture), duration, frequency, and severity of symptoms at baseline. We shall undertake a narrative synthesis if quantitative synthesis is not acceptable.

#### 2.5.4. Assessment of publication bias.

If >10 papers were included, funnel plots were used to measure publication bias. A symmetrical funnel plot data distribution suggests that there is no publishing bias.

#### 2.5.5. Grading the quality of evidence.

To assess the quality of evidence in this systematic review, the Grading of Recommendations Assessment, Development, and Evaluation criteria will be employed. And the evidence’s quality will be assessed as high, moderate, low, or extremely low.

### 2.6. Ethics and dissemination

There is no need for this study to obtain ethical approval because no participants’ private information would be included. This systematic review will be completed in accordance with the PRISMA principles. The results will be published in a peer-reviewed, open-access journal, as well as the completed systematic review. Furthermore, the meta-analysis will be disseminated online, where anyone can download it for free.

## 3. Discussion

The theoretical system of traditional Chinese medicine had evolved in the long course of clinical practice under the guidance of materialism and dialectics. It has originated from practice and, in turn, guides the practice. This unique system of theory is characterized by the conception of organic wholeness and treatment based on syndrome differentiation. The conception of organic wholeness refers to the unity and integrity the human body itself and its close relationship with the outer world. The unity of the body is realized through the dominant function of the heart with the assistance of the 6 Fu-organs and the meridians that “pertain to the viscera in the interior and connect with the limbs and joints in the exterior.” External therapy is an ever-widening presence within mainstream health care settings. The action of acupuncture with intervention by needling at the body surface affects the flow of qi in the meridians, thus balancing bodily disharmony.

The purpose of this systematic review is to assess the efficacy and safety of acupuncture in the treatment of IBD. Despite the fact that more and more research has been committed to IBD in recent years, there is still no gold standard for therapy. Acupuncture is becoming more prevalent in mainstream health care settings. The needles’ effect impacts the flow of qi in the meridians, thereby balancing body discord. Acupuncture has been shown in clinical studies to be effective in the treatment of IBD.^[25–29]^ However, a high-quality study has yet to be published. It is critical to carry out this technique in order to obtain meaningful results and deliver superior solutions.

This study has some advantages and limitations. This study will provide the first systematic review and meta-analysis for evaluating the efficacy and safety of acupuncture for IBD, which is designed according to the Preferred Reporting Items for Systematic reviews and Meta-Analyses Protocols (PRISMA-P) 2015 Statement. The protocol for this review has been registered in the PROSPERO network (No. CRD42021257520). Two review authors will select the studies, extract data and assess the risk of bias independently. However, the reliability of the results will largely depend on the methodological quality of primary studies included and the heterogeneity among them. There might be few studies with a low risk of bias; hence, they might affect the quality of the evidence.

## Author contributions

All authors have read and agreed to the published version of the manuscript.

**Conceptualization:** Xinyue Yang, Tie Li.

**Data curation:** Xinyue Yang, Qingqing Tang.

**Formal analysis:** Qingqing Tang.

**Funding acquisition:** Tie Li.

**Investigation:** Xinyue Yang, Qingqing Tang.

**Methodology:** Xinyue Yang.

**Project administration:** Zhihong Wang.

**Resources:** Qingqing Tang.

**Software:** Qingqing Tang.

**Supervision:** Zhihong Wang.

**Validation:** Mengmeng Sun, Min He.

**Visualization:** Qingqing Tang.

**Writing – original draft:** Xinyue Yang.

**Writing – review & editing:** Xinyue Yang, Mengmeng Sun, Min He.
